# Brain Dynamics and Connectivity from Birth through Adolescence

**DOI:** 10.3390/brainsci12030395

**Published:** 2022-03-15

**Authors:** Ardalan Aarabi

**Affiliations:** 1 Laboratory of Functional Neuroscience and Pathologies (LNFP, UR4559), University Research Center (CURS), University Hospital (CHU-Site Sud), 80054 Amiens, France; ardalan.aarabi@u-picardie.fr; 2 Faculty of Medicine, University of Picardy Jules Verne (UPJV), 80036 Amiens, France

The human brain as a complex dynamic system undergoes significant structural and functional changes from birth to adulthood to engender neurocognitive functions. In recent years, advances in neuroimaging techniques have allowed scientists to assess age-related changes in brain structure and organization and their relationship with cognitive measurements during healthy brain development. The assessment of the typical development of structural and functional brain networks also provides a baseline framework for better understanding disruptions in brain network topology and dynamics underlying cognitive impairment in neurological and neuropsychological diseases.

This Special Issue includes six articles advancing our understanding of alterations in brain network connectivity and dynamics in neurological and neuropsychological diseases employed especially in developmental neuroscience. These studies, briefly described below, demonstrate the experimental approaches highlighting novel findings on prematurity, cerebral palsy, traumatic brain injury (TBI), and attention-deficit-hyperactivity disorder (ADHD). The last article illustrates the usefulness of neurofeedback training for motor performance improvement, a method commonly used for the rehabilitation of patients with chronic stroke, schizophrenia, and Parkinson’s disease.

In the first article, Barnes-Davis et al. [1] assessed the relationship between functional hyperconnectivity and language attainments in extremely preterm (EPT, less than 28 weeks completed gestation) children. Premature birth is one of the leading causes of infant morbidity and mortality. Extreme prematurity is known to be associated with neurodevelopmental impairments including cognitive, motor, and language deficits. The authors investigated whether functional hyperconnectivity can be considered as a marker of language resiliency for EPT children. By investigating fMRI activation during story listening, the authors found that well-performing EPT and preterm children with a history of language delay were characterized with interhemispheric hyperconnectivity in bitemporal subnetworks in comparison with term children. Their findings suggest that hyperconnectivity might represent a marker of advancing language development, which could be used to target interventions and predict later functioning.

In the second article, Doucet et al. [2] assessed alterations in resting-state functional connectivity (FC) in the sensory and sensorimotor networks in youth with cerebral palsy (CP), which is the most prevalent pediatric neurologic impairment associated with mobility deficiencies, suggested to be associated with alterations in activities and functional connectivity of sensorimotor and visual cortices [2]. Using resting-state fMRI data, the authors found a characteristic loss of functional integration between the sensorimotor and visual networks in young patients with CP. Their results also revealed that higher FC between these networks was associated with larger step lengths in youth with CP. The authors conclude that altered FC between the motor and visual regions might play a role in the uncharacteristic mobility seen in youth with CP.

In the third article, Cao et al. [3] investigated alterations in the topological properties and dynamics of the functional brain networks involved in sustained attention processing in children with severe post-traumatic brain injury attention deficits (TBI-A) using task-based fMRI data. TBI is the leading cause of disability in children, often associated with neurocognitive deficits in attention, learning, and memory. The authors found lower degrees of functional integration in temporal and parietal regions during sustained attention processing in children with TBI-A compared to healthy controls. Their findings provide evidence concerning the temporal instability of the functional connectivity associated with TBI-related attention deficits in children. In the fourth article, the same research group [4] also examined alterations in the functional network organization and connectivity in both white matter (WM) and gray matter (GM) during sustained attention processing in adults with post-TBI attention deficits. The authors found significant alterations in functional integration and segregation in several WM and GM regions involved in visually sustained attention processing in the TBI group compared to healthy controls. Their findings emphasize the critical role of functional alterations associated with white matter tracts and their anatomically connected GM regions in post-TBI attention deficits.

In the fifth article, Ahmadi et al. [5] investigated alterations in brain functional connectivity in children with attention deficit hyperactivity disorder (ADHD), one of the most common childhood developmental disorders associated with impulsivity and/or difficulty in sustaining attention. The authors explored characteristic alterations in resting-state EEG source connectivity and the functional organization of so-called rich-club (cortical) hubs, associated with two clinically distinguishable inattentive and combined (characterized by both inattention and hyperactivity) ADHD subtypes. They found a reduced level of functional segregation and higher and lower levels of functional integration in low and high-frequency bands, respectively, for both subtypes compared to typically developing children. Their findings demonstrated characteristic frequency-specific functional reorganization of the brain networks associated with attention deficits and symptomatology in ADHD children.

The last article of this Special Issue focuses on the impact of the neurofeedback training of motor-related regions on the dynamic characteristics of FC underlying motor execution [6]. Neurofeedback is used to self-regulate brain activity in specific brain regions in order to improve cognitive and motor performance in patients with chronic stroke, schizophrenia, and Parkinson’s disease. Using real-time functional magnetic resonance imaging, Long and colleagues [6] identified a dynamic FC state exhibiting weak connections, found to be associated with the training effect and the improvement in motor performance. Overall, their findings show that neurofeedback can improve motor performance by changing the temporal properties of the dynamic functional network connectivity underlying motor execution tasks.

Overall, the articles in this Special Issue provide new insights into alterations in brain network connectivity and dynamics underlying neurological and neuropsychological diseases. We would like to thank all the authors and reviewers who have contributed to this Special Issue and the editorial staff for their outstanding support.
